# Adverse Cardiovascular Events Associated With Kopi Jantan

**DOI:** 10.7759/cureus.52344

**Published:** 2024-01-15

**Authors:** Mohd Khairi Othman, Zurkurnai Yusof, Nur Imani Mohd Rossli, Mohd Hafizazham Mohd Fauzi, W Yus Haniff W Isa

**Affiliations:** 1 Department of Internal Medicine, Universiti Sains Malaysia School of Medical Sciences, Kubang Kerian, MYS

**Keywords:** myocardial infarction, energy drink, acute coronary syndrome, atrial flutter with rapid ventricular response, myocardial infarction with non-obstructive coronary arteries (minoca)

## Abstract

Energy drinks (EDs) are widely accessible worldwide. In Malaysia, it is common for EDs to be premixed with sexual stimulants. ED consumption has been shown to have an association with cardiac arrest, myocardial infarction, spontaneous coronary artery dissection, and coronary vasospasm. In addition to this, EDs are associated with arrhythmias, which significantly prolong the QTc interval. Myocardial infarction with no obstructive coronary artery disease (MINOCA) is defined as a patient presenting with myocardial infarction with no obstructive coronary artery disease or ≤50% stenosis. It is a challenging and complex pathophysiology compared to obstructive coronary artery disease. MINOCA is more frequently associated with younger patients and women. Here, we report two cases related to a Malaysian local energy drink *Kopi Jantan*, which presented with atrial flutter and MINOCA.

## Introduction

There is a worldwide increase in the consumption of instant energy drinks (EDs) and supplements, particularly among adolescents and young adults [1]. The limited regulatory oversight and under-reported toxicity profile of these products reflect the constellation of newer adverse events. These drinks are aggressively marketed with the claim that they give an energy boost to improve physical and cognitive performance, especially in Malaysia. ED usage is reported to be increasing around the world, with 68% in adolescents and 30% in adults [2,3]. Excessive ED consumption has been shown to be associated with atrial arrhythmias, ventricular arrhythmias, QT interval prolongation, ST-elevation myocardial infarction, and increased blood pressure [1-4]. Caffeine stimulates and increases brain, nervous system, and cardiac activity. Caffeine is found in many drinks, such as coffee, tea, soft drinks, and EDs, and is associated with an increase in intracellular calcium concentration, which leads to a catecholamine surge resulting in supraventricular or ventricular arrhythmias [3,5]. Apart from caffeine, sildenafil is a common ingredient in the *Kopi Jantan* coffee mixture. One of the common side effects of excessive sildenafil usage is low blood pressure due to vasodilation.

## Case presentation

Case one

**Figure 1:**
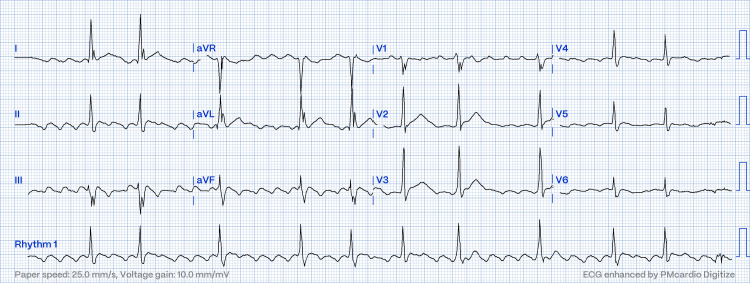
Electrocardiogram showing typical atrial flutter with a variable block (case one).

**Figure 2:**
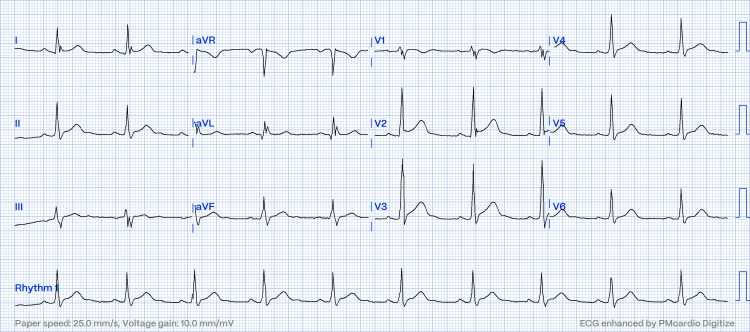
Electrocardiogram showing reversal to sinus rhythm post electrical cardioversion (case one).

A 33-year-old gentleman, married and an active smoker with underlying allergic rhinitis, presented to our center with a complaint of sudden-onset transient loss of consciousness while he was cleaning his house. The symptom was associated with urinary incontinence, chest discomfort, and palpitation. It was his first experience with these symptoms. On further questioning, the symptom started after consuming a local energy and sexual stimulant (Kopi Jantan) with instant premixed coffee for the past two years and increased in amount two months before this clinical presentation. He mainly took this drink for energy and sexual satisfaction. On examination, blood pressure (BP) was 140/80 mmHg, pulse rate was 120 beats per minute, and it was irregularly irregular. Physical examination was unremarkable. His electrocardiography (ECG) showed a typical counter-clockwise atrial flutter (Figure 1). Other blood investigations, including thyroid function tests, were within normal range. His urine toxicology for illicit drugs was negative. Echocardiography revealed a structurally normal heart chamber with preserved left ventricular ejection fraction (LVEF). A diagnosis of symptomatic atrial flutter with rapid ventricular response secondary to an ED was made. He was started on intravenous amiodarone, and synchronized cardioversion with 50 J was performed. The tachycardia terminated with a single synchronized cardioversion (Figure 2). He was admitted to the ward for two days for observation, and no arrhythmias were noted. Because his CHA2DS2VASc was 0, he was not started on an anticoagulant and discharged with tablet verapamil 40 mg TDS to maintain the sinus rhythm with outpatient ambulatory ECG follow-up.

Case two

A 27-year-old gentleman, a non-smoker with no traditional risk factors for atherosclerosis, presented with sudden-onset central chest pain, described as heaviness and crushing in nature, with a pain score of 10/10, radiating to the back. It was associated with profuse sweating and vomiting three times since the onset. Otherwise, there was no significant symptom. On further history taking, the patient reported consuming Kopi Jantan stimulant for two years for energy. Additionally, he had recently gotten married and was consuming Kopi Jantan more frequently than previously. Otherwise, there was no high-risk behavior or use of recreational drugs and or other traditional supplements or herbs. There was no history of sudden cardiac death, cardiovascular disease, or cardiomyopathy in the family.

**Figure 3:**
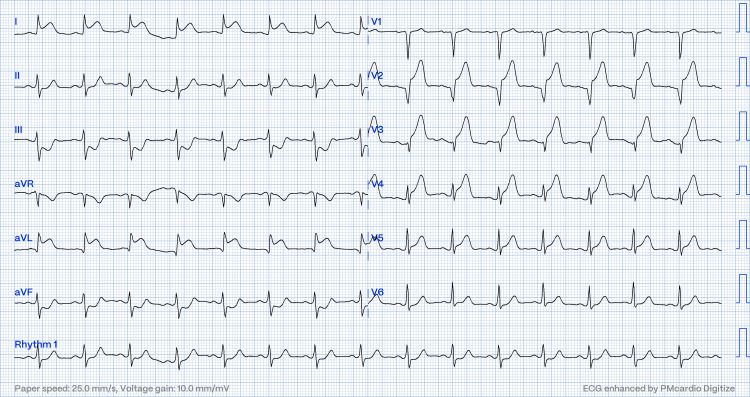
Electrocardiogram showing ST elevation at leads I, AVL, and V2-V4 with reciprocal changes (case two).

**Figure 4:**
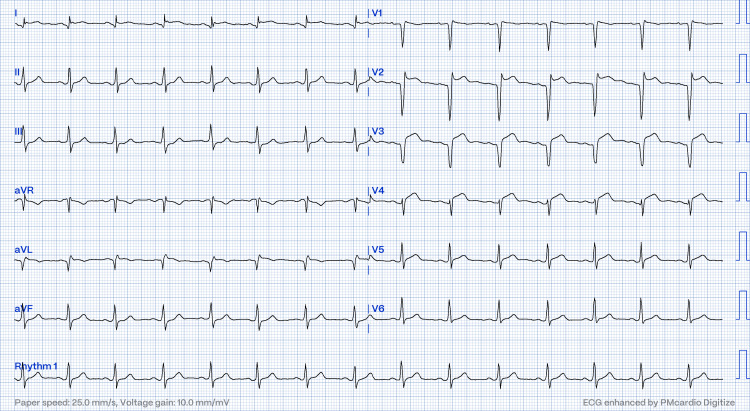
Post-thrombolysis electrocardiogram showing resolution of ST elevation in leads I, AVL, and V2-V4 (case two).

**Figure 5:**
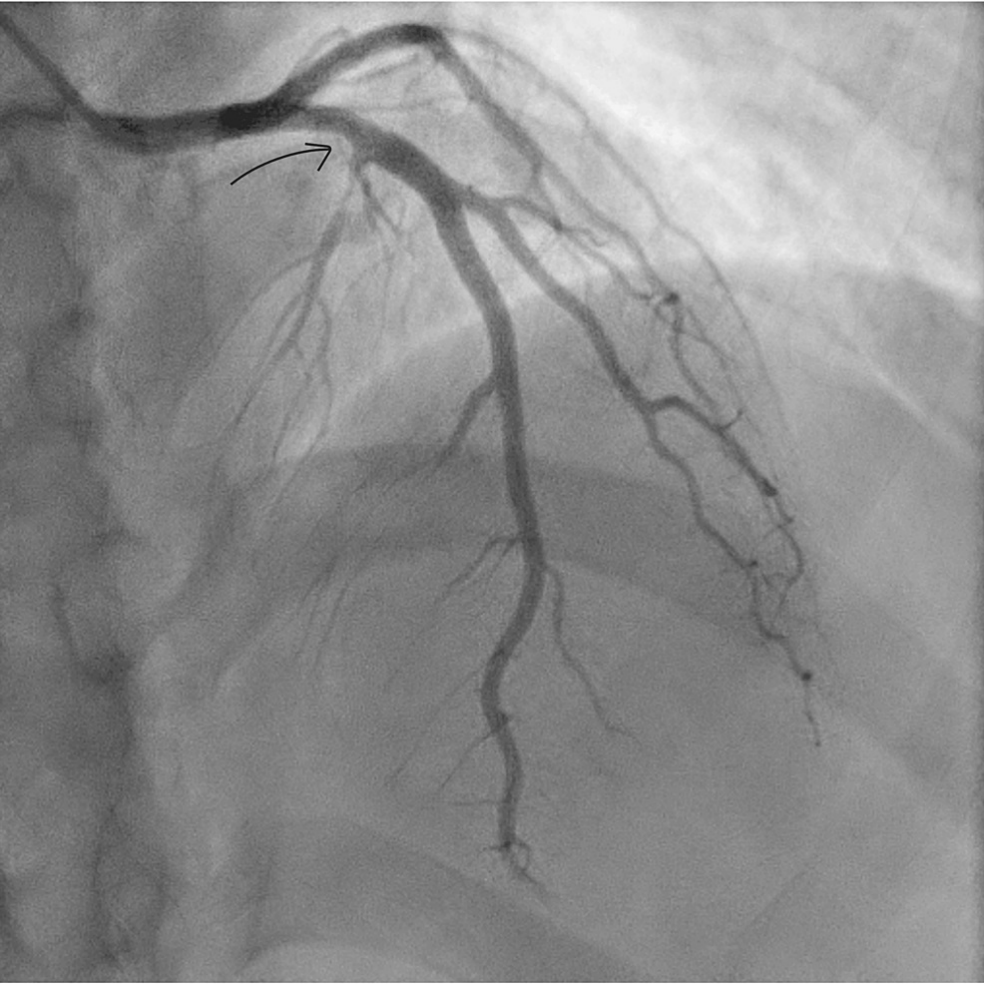
Anteroposterior cranial view of coronary angiography showing mild stenosis (labeled arrow) at the proximal left anterior descending artery (case two).

**Figure 6:**
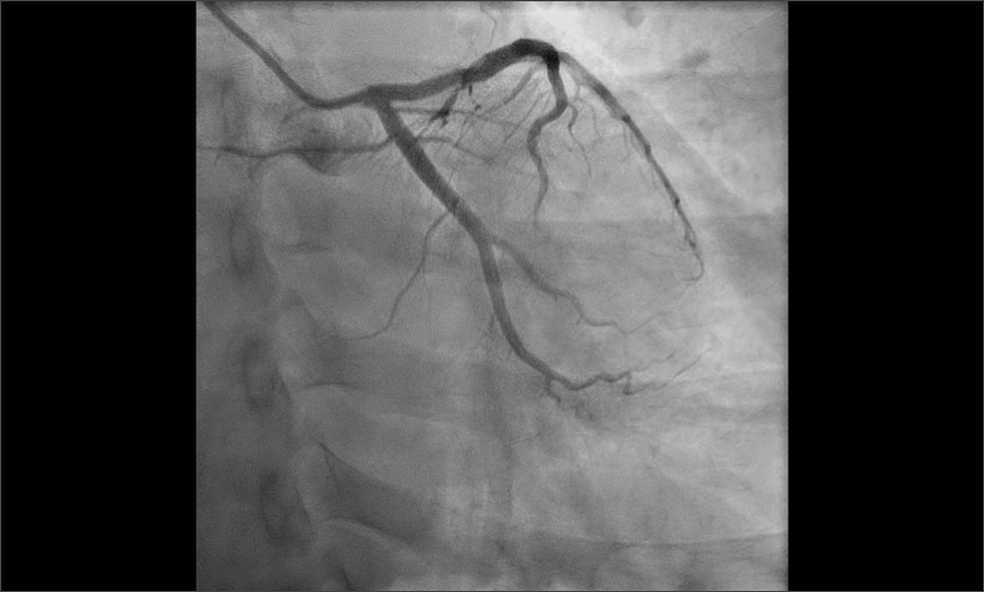
Anteroposterior caudal view of coronary angiography showing a normal left circumflex artery (case two).

**Figure 7:**
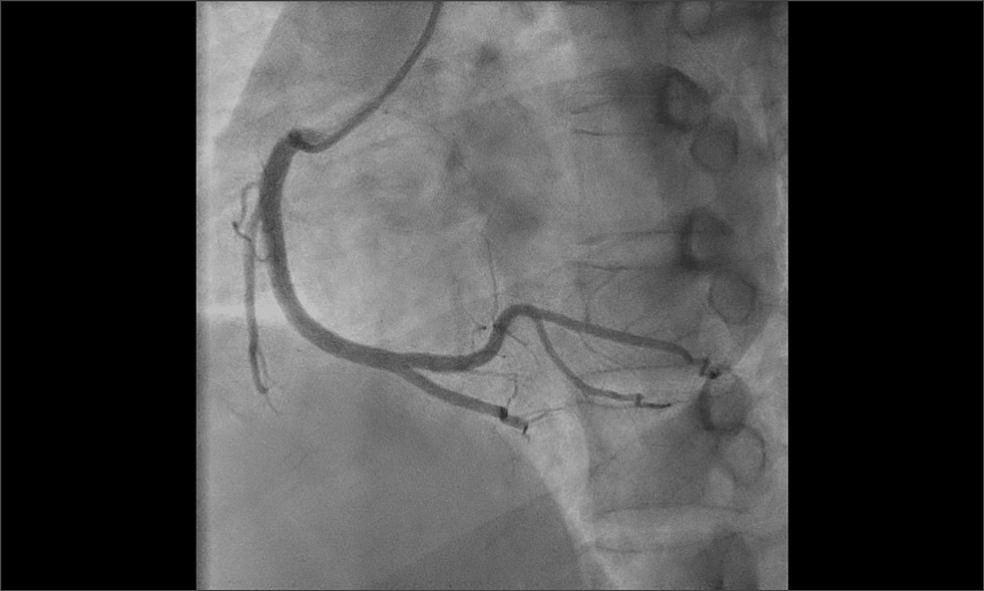
Anteroposterior cranial view of coronary angiography showing a normal right coronary artery (case two).

Upon presentation, his blood pressure was 141/96 mmHg, pulse rate was 92 beats per minute, and he was afebrile. Initial ECG showed ST elevation in leads I, AVL, and V2-V6 (Figure 3). He was successfully thrombolyzed with intravenous (IV) streptokinase 1.5 million units, and ECG showed resolution of ST-segment elevation (Figure 4). His blood investigation showed total white cells at 18.9 x 10^9^/L, hemoglobin at 16.9 g/dL, and platelet at 436 x 10^9^/L. Renal and liver function was within the normal range. His troponin T was 310 pg/L. Other investigations, such as urine toxicology, autoimmune disease, and infective screening, were negative. His echocardiography showed LVEF of 25% with hypokinesia anteroseptal, anterolateral, and lateral of the left ventricular wall with spontaneous echocardiographic contrast. Subsequently, coronary angiography was done during the same admission, which revealed non-obstructive coronary stenosis at the proximal left anterior descending artery, as shown in Figure 5. Other coronary vessels (left circumflex artery and right coronary artery) were normal (Figures 6, 7). Hence, he was diagnosed with myocardial infarction with non-obstructive coronary arteries (MINOCA) because of no significant obstructive coronary artery disease and acute thrombotic occlusion resolved with thrombolysis. We did not perform intravascular imaging due to financial constraints. He was discharged on dual antiplatelets and remained well on outpatient clinic follow-ups.

## Discussion

ED is a common beverage young adults consume for mental and physical stimulation [6]. The most common stimulant is caffeine. The rate of ED usage in Malaysia is comparable with the rest of the world. Mandilaras et al. (2022) reported the highest prevalence of ED consumption among adolescents (68%) [7]. Despite the association with adverse effects on the heart, the acceptance of such drinks is very high among them due to the good effect on their body physiology. Regarding caffeine usage, multiple studies have shown that excessive usage of caffeine is associated with tachyarrhythmias and myocardial infarction [5].

In Malaysia, Kopi Jantan is a local product labeled as traditional natural herbal coffee and is widely and easily available. The Food and Drug Administration has reported that sildenafil is its active ingredient on top of caffeine [8]. With two unknown doses of caffeine and sildenafil in each sachet, it can lead to potentially more harmful adverse effects on the heart, as shown in our patients. Sildenafil is commonly associated with hypotension, which was absent in our patients. Hypotension was not seen in both of our patients most likely due to the counter effect of a high dose of caffeine, which prevents hypotension. Both of our patients had recently married. Hence, the amount of this coffee and sildenafil combination was more for sexual stimulation. Caffeine acts as a proarrhythmic drug with biological plausibility associated with increased catecholamine levels and calcium release, possibly leading to delayed afterdepolarization [9-11]. Chronic caffeine usage is associated with tachyarrhythmias, especially atrial and supraventricular arrhythmias, due to prolonged left atrial effective refractory periods. It has been reported that caffeine overdose is associated with tachyarrhythmias due to beta-1 receptor stimulation [6]. Because our first patient did not have typical risk factors for atrial flutter, such as heart failure, congenital heart disease, rheumatic heart disease, and lung problems, the atrial flutter was most likely due to acute-on-chronic caffeine usage [12,13].

Apart from tachyarrhythmia, EDs are associated with myocardial infarction. A few studies have reported an association with excessive caffeine intake [14,15]. Excessive intake of Kopi Jantan associated with myocardial infarction has not been reported before. The postulated reason for ED associated with myocardial infarction is due to increased platelet aggregation and endothelial dysfunction leading to hypercoagulable state and thrombosis. Our patient had no traditional risk factors for myocardial infarction, and secondary causes of atherosclerosis were ruled out. His coronary angiography finding showed no obstructive coronary stenosis; hence, a diagnosis of MINOCA was made. MINOCA is characterized by normal or near-normal coronary artery on angiography [16].

Based on registries, the prevalence of MINOCA is between 5% and 25% [17]. MINOCA varies in etiologies and pathogenic mechanisms compared to obstructive myocardial infarction. In type 1 myocardial infarction, MINOCA comprises between 5% and 20%. A few mechanisms have been postulated for MINOCA, including coronary dissection, coronary artery spasm, microvascular coronary spasm, and coronary embolism [16]. Unfortunately, we did not perform intravascular imaging for our patient due to limited resources. We postulate that our second patient had an acute thrombotic occlusion, which responded to thrombolysis, and the coronary angiography did not show any significant coronary stenosis. In ED usage, studies have shown that there are significant increases in norepinephrine levels, which may predispose to cardiovascular risk [9,18]. Our patient received thrombolysis using IV streptokinase and underwent coronary angiography on a subsequent day due to a limitation of resources and staffing in our center to perform primary percutaneous coronary intervention.

Our cases add to the current literature on cardiovascular complications related to Kopi Jantan. It is a locally manufactured coffee drink easily available in Malaysia. Reporting these two cases having a temporal relationship with Kopi Jantan and cardiovascular side effects will help clinicians to be aware of this event.

## Conclusions

EDs are well known to be associated with adverse effects on the cardiovascular system, as reported in multiple literature reviews. Kopi Jantan is a local Malaysian ED with an unknown caffeine dosage and sildenafil per sachet. Excessive caffeine ingestion leads to cardiac arrhythmias and endothelial dysfunction. These cases illustrate the importance of carefully taking history for the temporal relationship of any ED upon encountering a young patient with an atypical presentation.

## References

[1] Pacheco Claudio C, Quesada O, Pepine CJ, Noel Bairey Merz C (2018). Why names matter for women: MINOCA/INOCA (myocardial infarction/ischemia and no obstructive coronary artery disease). Clin Cardiol.

[2] Shah SA, Szeto AH, Farewell R (2019). Impact of high volume energy drink consumption on electrocardiographic and blood pressure parameters: a randomized trial. J Am Heart Assoc.

[3] Oberhoffer FS, Li P, Jakob A, Dalla-Pozza R, Haas NA, Mandilaras G (2022). Energy drinks: effects on blood pressure and heart rate in children and teenagers. A randomized trial. Front Cardiovasc Med.

[4] Mangi MA, Rehman H, Rafique M, Illovsky M (2017). Energy drinks and the risk of cardiovascular disease: a review of current literature. Cureus.

[5] Grinberg N, Benkhedda K, Barber J, Krahn AD, La Vieille S (2022). Effects of caffeinated energy drinks on cardiovascular responses during exercise in healthy adults: a systematic review and meta-analysis of randomized controlled trials. Appl Physiol Nutr Metab.

[6] Kim EJ, Hoffmann TJ, Nah G, Vittinghoff E, Delling F, Marcus GM (2021). Coffee consumption and incident tachyarrhythmias: reported behavior, Mendelian randomization, and their interactions. JAMA Intern Med.

[7] Mandilaras G, Li P, Dalla-Pozza R, Haas NA, Oberhoffer FS (2022). Energy drinks and their acute effects on heart rhythm and electrocardiographic time Intervals in healthy children and teenagers: a randomized trial. Cells.

[8] Mo L, Xie W, Pu X, Ouyang D (2018). Coffee consumption and risk of myocardial infarction: a dose-response meta-analysis of observational studies. Oncotarget.

[9] Zhang Y, Kim C, Wasif N (2023). Alcohol and caffeine synergistically induce spontaneous ventricular tachyarrhythmias: ameliorated with dantrolene treatment. Heart Rhythm O2.

[10] Fabrizio C, Desiderio M, Coyne RF (2016). Electrocardiogram abnormalities of caffeine overdose. Circ Arrhythm Electrophysiol.

[11] Boyer M, Koplan BA (2005). Cardiology patient page. Atrial flutter. Circulation.

[12] Waldo AL (2000). Treatment of atrial flutter. Heart.

[13] Pallangyo P, Bhalia SV, Komba M, Mkojera ZS, Swai HJ, Mayala HA, Kisenge PR (2023). Acute myocardial infarction following the consumption of energy drink in a 28-year-old male: a case report. J Investig Med High Impact Case Rep.

[14] Wajih Ullah M, Lakhani S, Siddiq W, Handa A, Kahlon Y, Siddiqui T (2018). Energy drinks and myocardial infarction. Cureus.

[15] Raparelli V, Elharram M, Shimony A, Eisenberg MJ, Cheema AN, Pilote L (2018). Myocardial infarction with no obstructive coronary artery disease: angiographic and clinical insights in patients with premature presentation. Can J Cardiol.

[16] Scalone G, Niccoli G, Crea F (2019). Editor's choice- pathophysiology, diagnosis and management of MINOCA: an update. Eur Heart J Acute Cardiovasc Care.

[17] Grasser EK, Miles-Chan JL, Charrière N, Loonam CR, Dulloo AG, Montani JP (2016). Energy drinks and their impact on the cardiovascular system: potential mechanisms. Adv Nutr.

[18] (2024). Public notification: Kopi Jantan Tradisional Natural Herbs Coffee contains hidden drug ingredient. https://www.fda.gov/drugs/medication-health-fraud/public-notification-kopi-jantan-tradisional-natural-herbs-coffee-contains-hidden-drug-ingredient-0.

